# Medical M.P.s at Work

**Published:** 1897-10-09

**Authors:** 


### MEDICAL M.P.S AT WORK.

The House of Commons was recently saved by its medical members from being led into the commission of a serious blunder. At the last session of the Medical Council a memorandum was submitted which had been framed by the Society of Medical Officers of Health for Scotland, for the purpose of calling attention to provisions of the Bill as presented to Parliament, which, in the opinion of the society, would tend to the degradation of the public health service in that division of the kingdom. The Bill proposed that legal proceedings by local authorities, in abatement of conditions alleged to be dangerous or injurious to health, might in future be based on certificates granted by sanitary inspectors, who require no qualification either in medicine or in public health, and it also proposed that the qualifications in public health required of medical officers under the Burgh Police (Scotland) Act may in future be dispensed with, while it did not afford any sufficient safeguard against the misuse of the proposed relaxation of the law. The memorandum was referred by the Medical Council to a committee, of which Sir Richard Thorne-Thorne, K.C.B., was chairman, and which reported that they could not do otherwise than view with grave apprehension a proposal to place the opinion of the sanitary inspector, in matters where a judgment has to be formed as to what is or is not liable to constitute a danger to public health, on a statutory equality with that of the medical officer of health. In adopting this report, and in directing it to be forwarded to the Secretary for Scotland and to the Lord Advocate, the Medical Council called attention to the fact that the Legislature has from time to time extended the requirement that medical officers of health, before their appointment, should give definite evidence of the possession of acquirements fitting them for the discharge of their public duties, and that, if the opinions of officers, altogether without the requisite technical and professional training, were placed on a similar footing to those of medical officers of health, the efforts of the Council to ensure a distinctively high standard of proficiency in the public health service would be seriously frustrated.

On July 22nd, the Bill was under consideration in Committee of the House of Commons, when the proposal as to qualifications under the Police Act was withdrawn, and Captain Sinclair moved an amendment for the purpose of declaring the subordination of the other sanitary officers to the medical officer of health; but this was opposed by the Lord Advocate, on the ground that the feeling of the Special Committee on Scotch Bills had been that the local authority should be the head, and that the medical officers should be their servants; while, if the amendment were carried, the medical officers would practically be placed over the local authorities. This was presumably argument, although it certainly was not reasoning; but it sufficed for the purpose, and the amendment was lost. On the special point raised by the Medical Council the Lord Advocate was also disposed to give trouble, and moved an amendment which would have amended nothing, and have left the evils pointed out by the Council in unchecked operation. At this point Sir William Priestley came to the rescue, and moved the omission of the whole of the sentence on which the question had arisen. Sir William pointed out that a medical certificate on the subject of a nuisance was an important matter, and if the sanitary inspector were allowed to certify in exactly the same way as a medical officer, it would be derogatory to the medical profession and often troublesome to the public. By omitting the proviso about a certificate entirely the Scottish practice on the subject would be assimilated to that of England, which worked exceedingly well. To this arrangement the Lord Advocate, apparently not with a very good grace, consented, after it had been supported by Sir Walter Foster, and opposed by Mr. Hedderwick on the strange ground that the unanimity of the medical profession rendered him suspicious. It would seem as if a certain section of the less intelligent politicians will never learn that the whole of the effective knowledge which we possess with regard to sanitary requirements, and to the means of preserving health, has been the free gift of the medical profession to the public, and that the profession stands alone among human callings in its persistent endeavour to control the evils by which it lives, and to diminish the necessity for its own services. The unanimity of the ignorant might in many cases be suspicious; but unanimity on the part of the possessors of knowledge should be regarded by all reasonable people as a perfectly secure basis of action.

